# Supplementary material for: A New Exopolysaccharide of Marine Coral-Associated Aspergillus pseudoglaucus SCAU265: Structural Characterization and Immunomodulatory Activity
Source: J Fungi (Basel). 2023 Oct 27;9(11):1057. doi: 10.3390/jof9111057 (PMC10672155; doi:10.3390/jof9111057)
Supplement: Supplementary file 1 [file jof-09-01057-s001.zip › jof-2615601-supplementary.pdf]

A new exopolysaccharide of marine coral-associated *Aspergillus* sp. SCAU265: Structural characterization and immunomodulatory activity

Bo Peng[1†], Yongchun Liu[2†], Yuqi Lin[3], Supaluck Kraithong[3], Li Mo[2], Ziqing Gao[2], Riming Huang [3*], Xiaoyong Zhang[2*]

[1]Institute for Environmental and Climate Research, Jinan University, Guangzhou, 511443, China

[2]University Joint Laboratory of Guangdong Province, Hong Kong and Macao Region on Marine Bioresource Conservation and Exploitation, college of Marine Sciences, South China Agricultural University, Guangzhou, Guangdong, 510642, China

[3]Guangdong Provincial Key Laboratory of Nutraceuticals and Functional Foods, College of Food Science, South China Agricultural University, Guangzhou 510642, China

[†]These authors contributed equally to this work

*Correspondence: huangriming@scau.edu.cn (R. H.); zhangxiaoyong@scau.edu.cn (X. Z.)

Table S1. Significant differences in metabolites between the ASP-1 treatment group and the control group

Metabolites	Class	VIP	FC	*p*-value
L-threo-3-Methylmalate	Alcohols and polyols	1.6485	8.66	< 0.0001
IMP	Alcohols and polyols	1.6359	2.2	0.0001
Phosphohydroxypyruvic acid	Alpha-keto acids and derivatives	1.4011	1.05	0.0327
Porphobilinogen	Amines	1.4694	0.73	0.0014
Citrulline	Amino acids, peptides, and analogues	1.5132	86.22	< 0.0001
N-Acetylglutamic acid	Amino acids, peptides, and analogues	1.6465	1.93	< 0.0001
L-Glutamic acid	Amino acids, peptides, and analogues	1.6408	1.59	< 0.0001
Dimethylglycine	Amino acids, peptides, and analogues	1.636	1.54	0.0001
Asymmetric dimethylarginine	Amino acids, peptides, and analogues	1.4012	0.75	0.0083
L-Asparagine	Amino acids, peptides, and analogues	1.5852	0.73	0.0022
L-Serine	Amino acids, peptides, and analogues	1.6091	0.72	0.0009
L-Aspartic acid	Amino acids, peptides, and analogues	1.6459	0.42	< 0.0001
Creatinine	Amino acids, peptides, and analogues	1.355	0.36	0.0162
L-Isoleucine	Amino acids, peptides, and analogues	1.2413	0.34	0.0465
(2E)-Decenoyl-ACP	Amino acids, peptides, and analogues	1.4647	0.2	0.0017
Metoclopramide	Aminophenyl ethers	1.2462	0.68	0.0455
Dehydroepiandrosterone	Androstane steroids	1.2769	0.09	0.0352
Salicylic acid	Benzoic acids and derivatives	1.5322	0.2	0.036
Phthalic acid	Benzoic acids and derivatives	1.3903	0.09	0.0352
D-Galactose	Carbohydrates and carbohydrate conjugates	1.4425	3.83	0.0234
trans-1,2-Cyclohexanediol	Carbohydrates and carbohydrate conjugates	1.5167	3.37	0.0092
N-Acetyl-a-neuraminic acid	Carbohydrates and carbohydrate conjugates	1.5913	0.44	0.0018
Sorbitol	Carbohydrates and carbohydrate conjugates	1.2954	0.27	0.0307
Mannitol	Carbohydrates and carbohydrate conjugates	1.3061	0.23	0.0266
Cholesterol	Cholestane steroids	1.4685	0.02	0.0014
Cyclic GMP	Cyclic purine nucleotides	1.2344	2.76	0.0472
Succinic acid	Dicarboxylic acids and derivatives	1.6484	4.73	< 0.0001
19(S)-HETE	Eicosanoids	1.3466	1.29	0.0475
Propionylcarnitine	Fatty acid esters	1.4458	0.73	0.0031
Itaconic acid	Fatty acids and conjugates	1.6485	21.58	< 0.0001
Undecanoic acid	Fatty acids and conjugates	1.3036	4.09	0.0276
Palmitic acid	Fatty acids and conjugates	1.3456	3.23	0.0464
7,8-Diaminononanoate	Fatty acids and conjugates	1.5015	2.19	0.0001
Docosapentaenoic acid (22n-3)	Fatty acids and conjugates	1.5057	0.85	0.0107
5-Guanidino-3-methyl-2-oxopentanoate	Fatty acids and conjugates	1.2722	0.78	0.0365
Palmitoleic acid	Fatty acids and conjugates	1.3873	0.53	0.0106
Glycerophosphocholine	Glycerophosphocholines	1.4362	2.18	0.004
Gamma-Linolenic acid	Lineolic acids and derivatives	1.4481	2.55	0.003
Oxoadipic acid	Medium-chain keto acids and derivatives	1.6461	4.38	< 0.0001
3-Carbamoyl-2-phenylpropionaldehyde	Phenylacetaldehydes	1.394	1.34	0.0095
O-Phosphoethanolamine	Phosphate esters	1.3125	0.34	0.0256
Pantothenic acid	Polyols	1.597	1.25	0.015
Pterin	Pterins and derivatives	1.5008	2.61	0.0001
1-Methyladenosine	Purine nucleosides	1.5033	0.34	0.0001
Xanthylic acid	Purine ribonucleotides	1.431	0.56	0.0244
Xanthine	Purines and purine derivatives	1.5746	0.85	0.0031
Pyridoxal 5'-phosphate	Pyridine carboxaldehydes	1.4346	0.61	0.0043
Quinolinic acid	Pyridinecarboxylic acids and derivatives	1.4899	2.63	0.0004
Pyridoxine	Pyridoxines	1.3949	0.63	0.0093
Uridine	Pyrimidine nucleosides	1.6407	1.41	< 0.0001
Uridine diphosphate-N-acetylglucosamine	Pyrimidine nucleotide sugars	1.472	0.33	0.0173
Cytosine	Pyrimidines and pyrimidine derivatives	1.4032	1.41	0.0079
Cytidine	Pyrimidines and pyrimidine derivatives	1.44	1.4	0.0036
Aprobarbital	Pyrimidines and pyrimidine derivatives	1.6484	0.25	< 0.0001
L-Carnitine	Quaternary ammonium salts	1.4704	0.54	0.0013
Fumaric acid	Short-chain keto acids and derivatives	1.6191	0.81	0.0005
(2R)-2-Hydroxy-3-(phosphonatooxy)propanoate	Sugar acids and derivatives	1.5688	1.2	0.0035
Citric acid	Tricarboxylic acids and derivatives	1.6378	1.92	0.0001
Phenyl acetate	others	1.5735	0.34	0.0029
2-Deoxystreptamine	others	1.2444	0.75	0.0442
2-Acetamidofluorene	others	1.3594	0.68	0.0155
2-Iminobutanoate	others	1.2298	0.67	0.049
NAD	others	1.3246	0.3	0.0226

VIP: Variable importance in projection, FC: Fold Change

Table S2. Up- or down-regulated metabolites in the top 20 enrichment pathways (The ones marked in red are amino acids)

Pathway (Top 20 enrichment)	Up metabolites	Down metabolites
Central carbon metabolism in cancer	L-Glutamic acid; Succinic acid; L-Aspartic acid; L-Serine	Fumaric acid; L-Asparagine; Citric acid; (2R)-2-Hydroxy-3-(phosphonatooxy)propanoate; L-Isoleucine
Biosynthesis of plant secondary metabolites	L-Glutamic acid; Succinic acid; L-Aspartic acid; L-Serine; Fumaric acid; IMP; L-Asparagineacid	Citric acid; (2R)-2-Hydroxy-3-(phosphonatooxy) propanoate; Palmitic acid; Xanthine; L-Isoleucine; Xanthylic acid; Quinolinic acid
Biosynthesis of alkaloids derived from histidine and purine	Succinic acid; Fumaric acid; IMP; Citric acid	(2R)-2-Hydroxy-3-(phosphonatooxy)propanoate; Xanthine; Xanthylic acid
Biosynthesis of amino acids	L-Glutamic acid; L-Aspartic acid; L-Serine; L-Asparagine; Citric acid; (2R)-2-Hydroxy-3-(phosphonatooxy) propanoate; Oxoadipic acid	Citrulline; L-Isoleucine; N-Acetylglutamic acid; Phosphohydroxypyruvic acid
Alanine, aspartate and glutamate metabolism	L-Glutamic acid; Succinic acid; L-Aspartic acid	Fumaric acid; L-Asparagine; Citric acid
Biosynthesis of alkaloids derived from ornithine, lysine and nicotinic acid	L-Glutamic acid; Succinic acid; L-Aspartic acid; Fumaric acid; Citric acid	(2R)-2-Hydroxy-3-(phosphonatooxy) propanoate; L-Isoleucine; Quinolinic acid
Arginine biosynthesis	L-Glutamic acid; L-Aspartic acid; Fumaric acid	Citrulline; N-Acetylglutamic acid
Biosynthesis of cofactors	NAD; Pyridoxal 5'-phosphate; L-Glutamic acid; L-Aspartic acid; L-Serine; IMP; Citric acid; (2R)-2-Hydroxy-3-(phosphonatooxy) propanoate	Pyridoxine; Oxoadipic acid; Pantothenic acid; Porphobilinogen; 7,8-Diaminononanoate; Quinolinic acid
Biosynthesis of plant hormones	Succinic acid; L-Aspartic acid; Fumaric acid; IMP	Citric acid; (2R)-2-Hydroxy-3-(phosphonatooxy)propanoate; Salicylic acid
ABC transporters	L-Glutamic acid; L-Aspartic acid; L-Serine	Uridine; Mannitol; L-Isoleucine; Cytidine; Sorbitol; Phthalic acid
Carbon metabolism	L-Glutamic acid; Succinic acid; L-Aspartic acid; L-Serine; Fumaric acid	Citric acid; (2R)-2-Hydroxy-3-(phosphonatooxy) propanoate; Phosphohydroxypyruvic acid
Biosynthesis of various secondary metabolites - part 3	L-Glutamic acid; L-Aspartic acid; L-Serine; Citric acid	(2R)-2-Hydroxy-3-(phosphonatooxy) propanoate; Citrulline
Protein digestion and absorption	L-Glutamic acid	L-Aspartic acid; L-Serine; L-Asparagine; L-Isoleucine
Biosynthesis of alkaloids derived from terpenoid and polyketide	Succinic acid; Fumaric acid; Citric acid	Cholesterol; (2R)-2-Hydroxy-3-(phosphonatooxy) propanoate
Glucagon signaling pathway	Succinic acid; Fumaric acid; Citric acid	(2R)-2-Hydroxy-3-(phosphonatooxy) propanoate
Glycine, serine and threonine metabolism	L-Aspartic acid; L-Serine; (2R)-2-Hydroxy-3-(phosphonatooxy) propanoate; Dimethylglycine; Phosphohydroxypyruvic acid	Dimethylglycine; Phosphohydroxypyruvic acid
Aminoacyl-tRNA biosynthesis	L-Glutamic acid	L-Aspartic acid; L-Serine; L-Asparagine; L-Isoleucine
Two-component system	L-Glutamic acid; Succinic acid; L-Aspartic acid	Fumaric acid; Citric acid
Mineral absorption	L-Serine	D-Galactose; L-Asparagine; L-Isoleucine
Nicotinate and nicotinamide metabolism	NAD; Succinic acid	L-Aspartic acid; Fumaric acid; Quinolinic acid

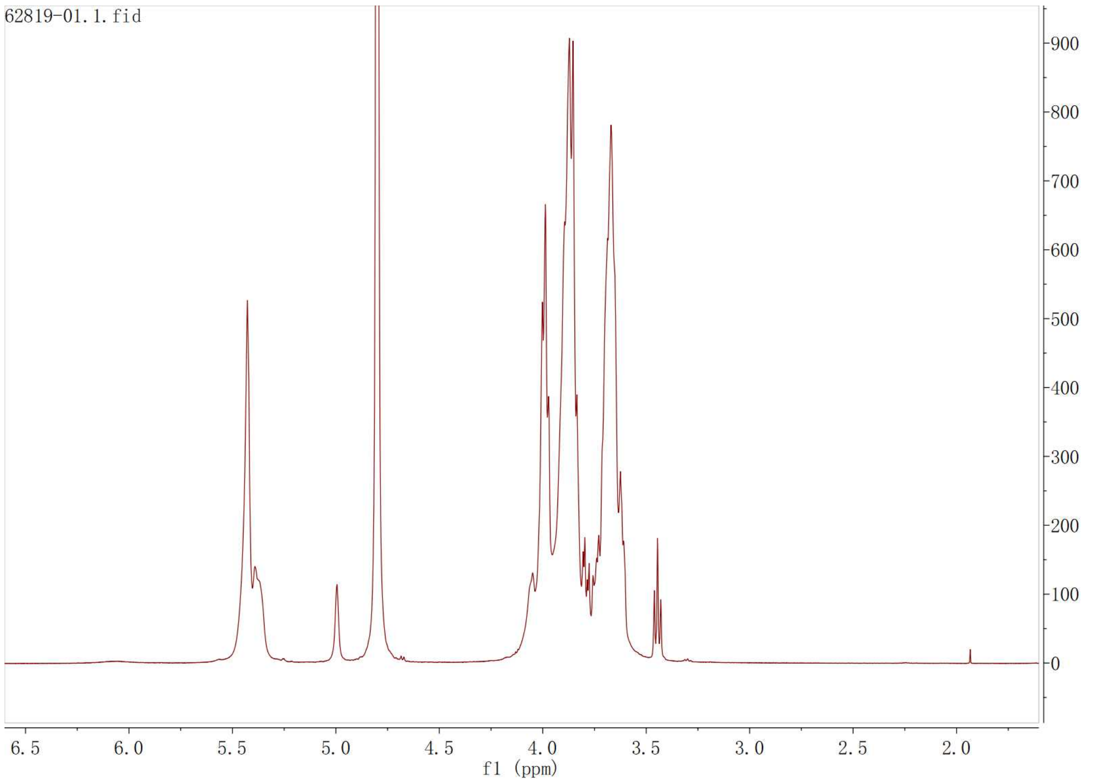

Figure S1. The original image (data) of 1H NMR of ASP-1

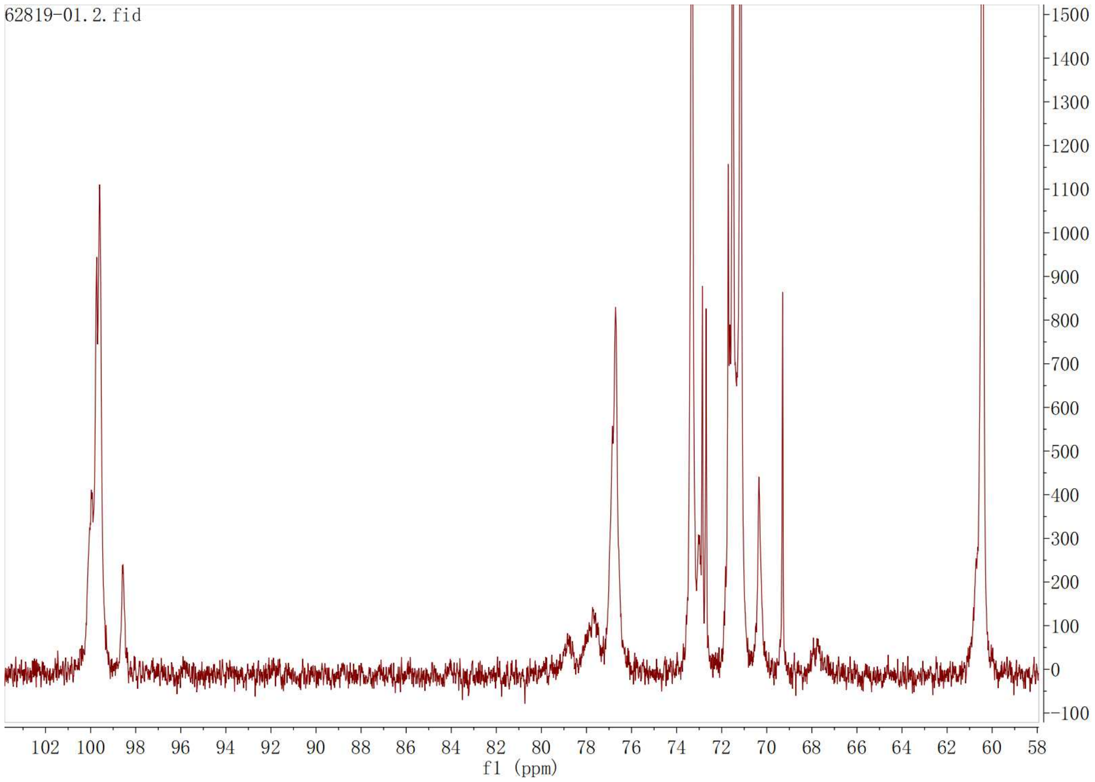

Figure S2. The original image (data) of 13C NMR of ASP-1

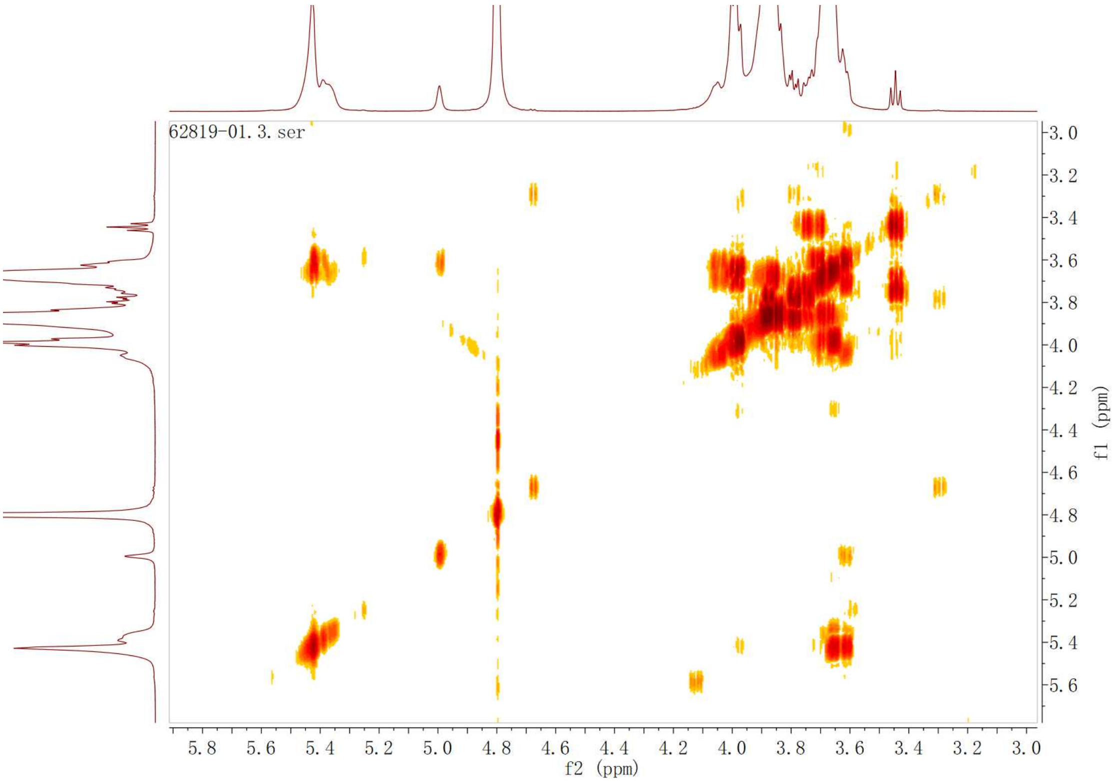

Figure S3. The original image (data) of 1H-1H COSY of ASP-1

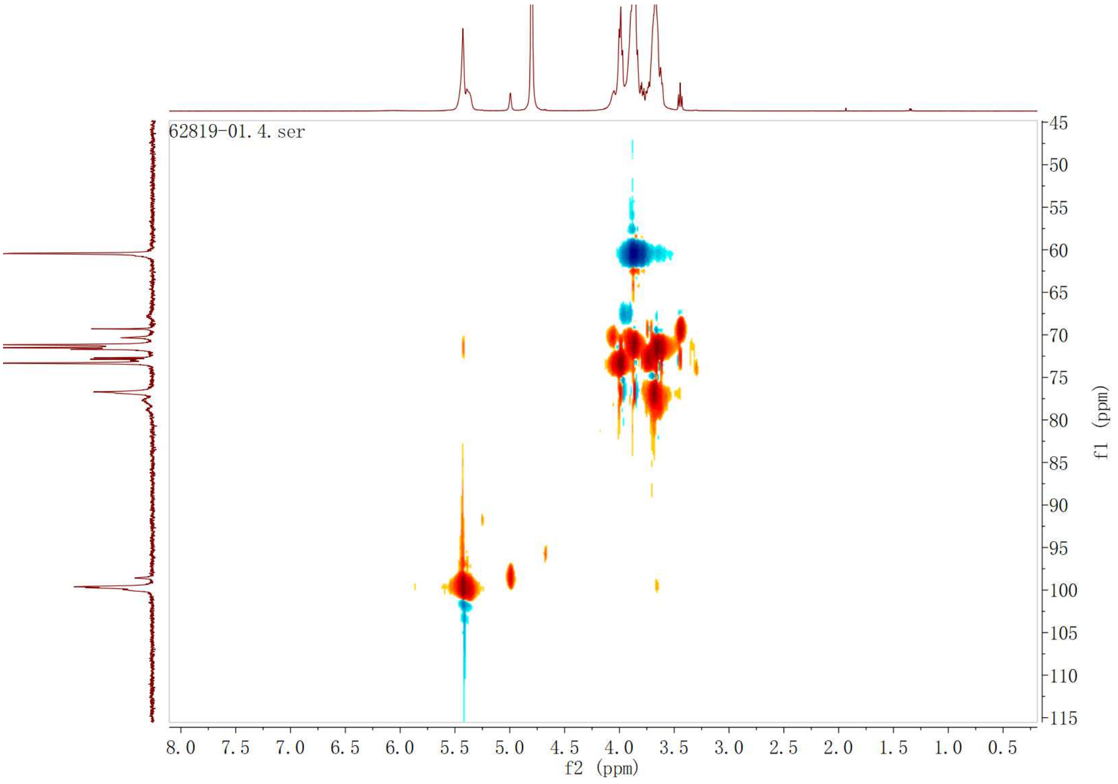

Figure S4. The original image (data) of HSQC of ASP-1

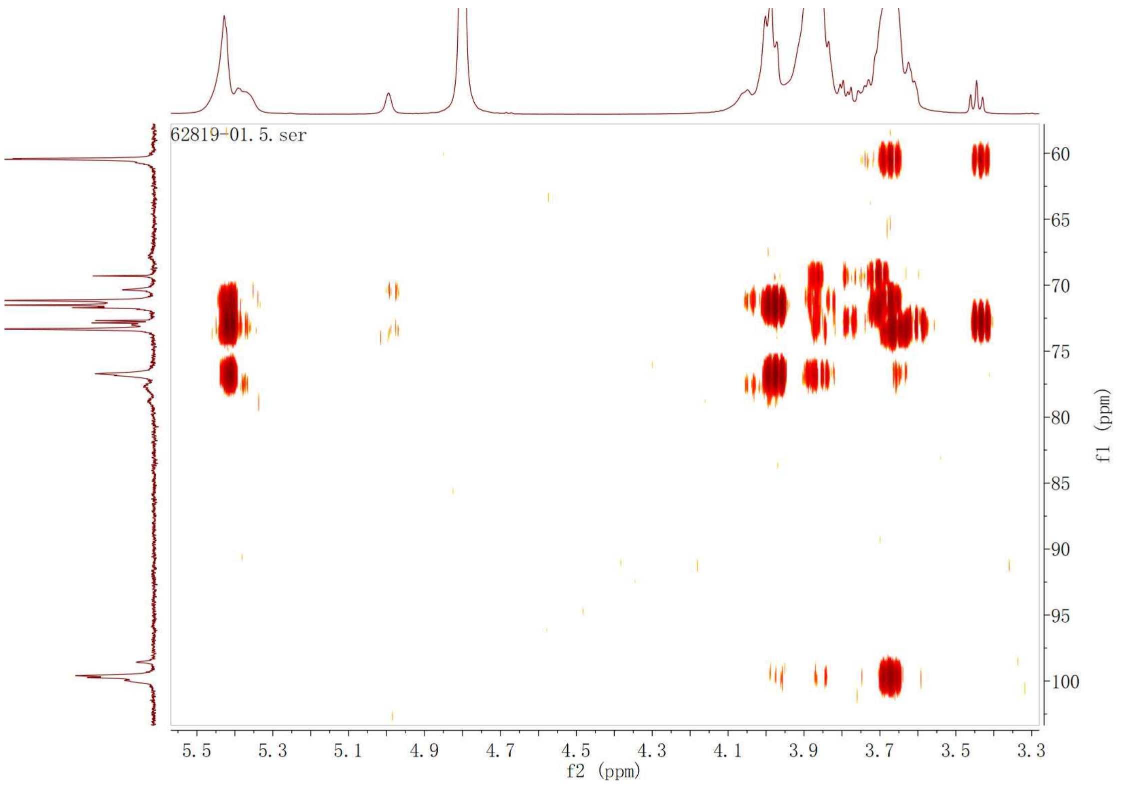

Figure S5. The original image (data) of HMBC of ASP-1

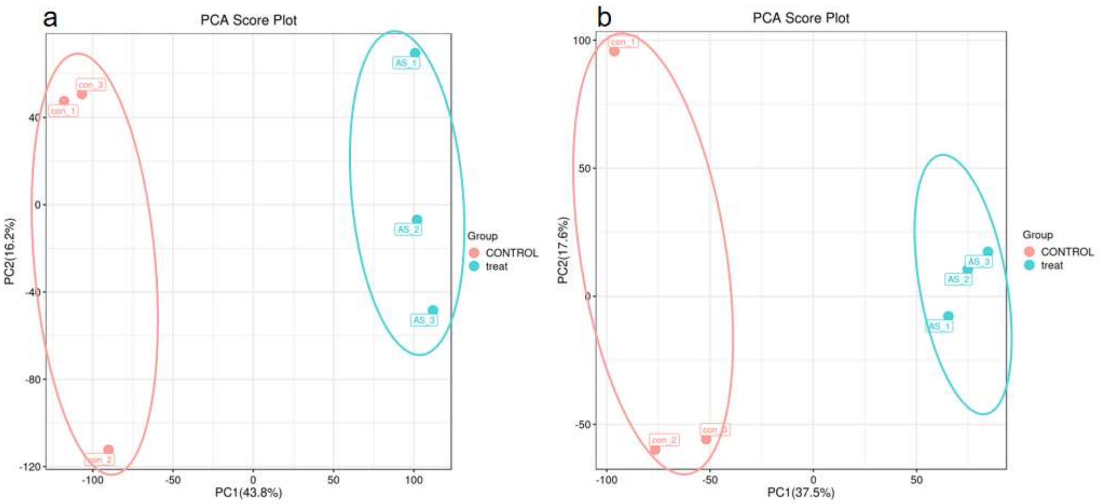

Figure S6. Principal Component Analysis (PCA) score plot of the macrophage RAW 264.7 cell samples from ASP-1 treatment and control groups in positive (a) and negative (b) ion modes. Con_1, 2 and 3 represent three parallel control samples, and AS_1, 2 and 3 represent three parallel treated samples with ASP-1.

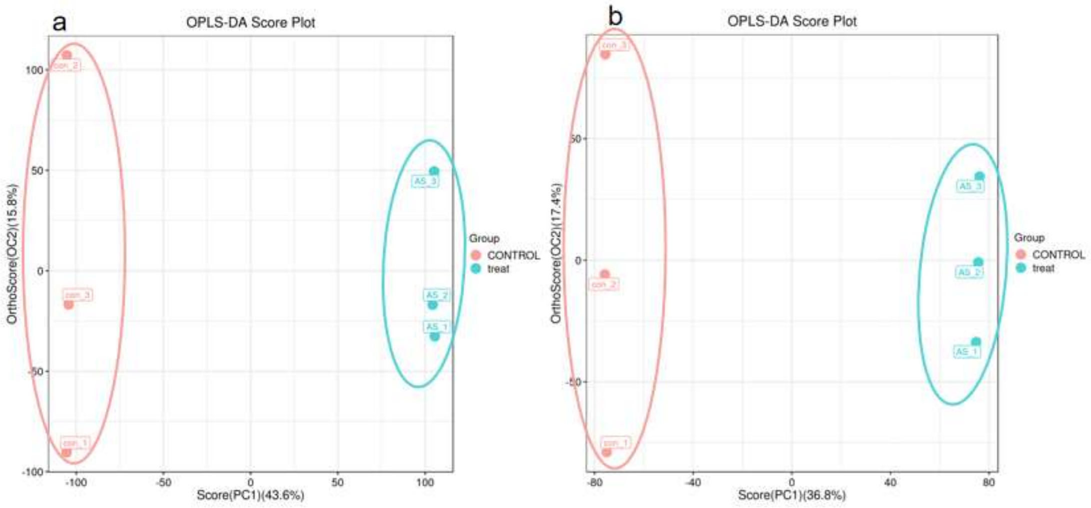

Figure S7. Orthogonal paarrtial least-squares discriminant analysis (OPLS-DA) score plot of the macrophage RAW 264.7 cell samples from ASP-1 treatment and control groups in positive (a) and negative (b) ion modes. Con_1, 2 and 3 represent three parallel control samples, and AS_1, 2 and 3 represent three parallel treated samples with ASP-1.
